# Circulation of ESBL-producing *Klebsiella pneumoniae* at the human–animal–environment interface in farms on the central coast of Peru

**DOI:** 10.3389/fpubh.2026.1736106

**Published:** 2026-02-05

**Authors:** Julio A. Benavides, Marília Salgado-Caxito, Patricia Escobar-Páramo, Daphne Léon, Luis M. Jara, Olga Bardales-Mendoza, Clara Murga, Patricia Medrano, Veronica Pérez, Brenda Aylas-Jurado, Roberto Su-Tello, Juana Najarro, Raiza Osorio-Linares, Elías Salvador-Tasayco, Carlos Shiva

**Affiliations:** 1 UMR MIVEGEC, IRD, CNRS, University of Montpellier, Montpellier, France; 2 One Health Institute and Doctorado en Medicina de la Conservación, Faculty of Life Sciences, Universidad Andrés Bello, Santiago, Chile; 3 Facultad de Medicina Veterinaria y Zootecnia, Universidad Peruana Cayetano Heredia, Lima, Peru; 4 Facultad de Educación, Universidad Peruana Cayetano Heredia, Lima, Peru; 5 Emerge, Unidad de Investigación en Enfermedades Emergentes y Cambio Climático, Facultad de Salud Pública y Administración, Universidad Peruana Cayetano Heredia, Lima, Peru; 6 Facultad de Medicina Veterinaria y Zootecnia, Universidad Nacional San Luis Gonzaga, Ica, Peru

**Keywords:** antimicrobial resistance, domestic animal, extended-spectrum *β*-lactamase, farmer, Latin America, livestock, One Health, water

## Abstract

Antibiotic-resistant bacteria, such as Extended-spectrum *β*-lactamase-producing *Klebsiella pneumoniae* (ESBL-*Kp*), represent a major threat to public health. Despite increasing reports of community-acquired ESBL-*Kp* infections, the dynamics of ESBL-*Kp* in low- and middle-income countries communities remains poorly understood. This study aimed to detect the fecal carriage of ESBL-*Kp* at the human–animal–environment interface in Peru and to characterize underlying molecular mechanisms involved. We detected 15 ESBL-*Kp* isolates among 652 (2.3%) fecal and water samples (i.e., 244 dairy cows from 25 farms, 261 pigs from 25 farms, 58 dogs, 39 farmers, and 50 water sources). ESBL-*Kp* was isolated from two humans, two dogs, four cows, three pigs, and four water sources. Genomic analyses identified 10 different ESBL-*Kp* sequence types (STs), including high-risk clones ST11, ST307, and ST37, as well as ST45, ST111, ST1, ST348, ST789, ST881, and ST983, and three CTX-M-encoding genes (*bla_CTX-M-15_*, *bla_CTX-M-27_*, *bla_CTX-M-14_*). Four ESBL-*Kp* STs (ST348, ST983, ST789, and ST11) were found in more than one source, both within and between farms. In particular, two ESBL-*Kp* ST983 isolates, one from a human and one from a cow on the same farm, differed by 37 SNPs and had almost identical genetic profiles, suggesting bacterial clonal exchange across host species or contamination from a common source. Likewise, two nearly identical ST348 isolates were recovered from a cow and a water source on the same farm, suggesting ESBL-*Kp* exchange between livestock and water. Our results highlight the circulation of ESBL-*Kp* across humans, animals, and water in rural environments in Peru, representing one of the first genomic studies exploring potential cross-species ESBL-*Kp* relatedness in Latin America. Our study supports the need to implement sanitary measures to limit the cross-species spread of antimicrobial-resistant bacteria and to reduce water source contamination in Peru.

## 1 Introduction

Extended-spectrum β-lactamase-producing *Klebsiella pneumoniae* (ESBL-*Kp*) represents an important threat to public health, and has been traditionally regarded as a hospital-associated pathogen (1). However, growing evidence suggests that ESBL-*Kp* can also be acquired in community settings, highlighting the need to understand its circulation within the community (2, 3). *Klebsiella pneumoniae* (*Kp*) is naturally present in the microbiome of humans and animals, where it can also act as an opportunistic nosocomial pathogen, and is estimated to cause approximately one-third of all Gram-negative bacterial infections (4). *Kp* can cause a wide range of diseases including pneumonia, urinary tract infections (UTIs), cystitis, bloodstream infections, and septicemia (5). The rise of multidrug-resistant *Kp*, particularly ESBL-*Kp*, poses a serious public health threat due to limited therapeutic options available, as well as its associated increases in mortality, hospital length of stay, and healthcare costs (5, 6).

ESBL-*Kp* circulates in hospital settings at variable levels across regions. The prevalence of hospital-acquired infections in high-income countries (HICs) ranges from 8% in Australia and New Zealand, 12% in the USA, 13% in Canada, and up to 21% in Western Europe (7). In contrast, low- and middle-income countries (LMICs) report higher prevalence, ranging from 23% in Asia excluding China, 32% in Eastern Europe, and up to 40% in the Middle East and Africa (7). Latin America has also reported high hospital ESBL-*Kp* prevalence ranging from 40 to 60% (8–10). ESBL-*Kp* also causes community-acquired infections, including pneumonia, UTIs, and bacteremia (11–13). Understanding the dynamics of ESBL-*Kp* in community settings is essential to mitigate its burden. For example, a high prevalence of ESBL among community-acquired *Kp* infections has been estimated in LMICs including Asian countries (e.g., 17% in bloodstream infections in China (14); 10–15% in UTIs in India and Iran (15, 16)). The estimated proportion of ESBL-*Kp* in community-acquired *Kp* infections in Latin America is high compared to other regions, ranging from 18% in community-acquired UTIs caused by *Kp* in Cuba (17), to 37% in community-acquired bloodstream infections caused by *Kp* in Brazil (18). Thus, understanding the dynamics of ESBL-*Kp* in Latin American communities can contribute to reducing its burden.

Several studies have reported the fecal carriage of ESBL-*Kp* in healthy human populations. For example, a prevalence of <1% of ESBL-*Kp* has been detected in healthy humans of HICs [e.g., Spain, France, Sweden, and Norway (19)]. In contrast, the limited data available from LMICs suggest higher level of community intestinal carriage of ESBL-*Kp* in African and Asian countries [e.g., India: 6% (20); Ethiopia: 6% (21); Nepal: 10% (22); Chad: 13% (23)], while there is a lack of epidemiological data on the human fecal carriage of ESBL-*Kp* in Latin America. *Kp* can also colonize animals (24–26) and persist or disseminate through water sources (27), underscoring the need for integrated One Health approaches to study the spread of ESBL-*Kp* in the community. However, the transmission dynamics of ESBL-*Kp* at the human–animal–environment interface are poorly understood (28). In rural areas of Latin America, inadequate sanitation and inappropriate antibiotic use are widespread (29, 30), potentially favoring the selection and dissemination of ESBL-*Kp* across the human–animal–environment interface.

Bacterial genomic analyses can contribute to identifying the genetic mechanisms driving ESBL-*Kp* circulation in the community (e.g., *Kp* clones, mobile genetic elements), as well as elucidating transmission pathways. A diverse pool of ESBL-*Kp* sequence types (STs) has been reported to carry different ESBL-encoding genes (e.g., CTX-M, SHV, TEM) worldwide (31). However, hospital-acquired ESBL-*Kp* infections are still dominated by high-risk clones, including ST307, ST15, ST147, ST405, and ST11, usually encoding *bla_CTX-M-15_* on plasmids (e.g., IncFIB/IncFII plasmid replicons) (32, 33). Latin America also reflects a dominance of *Kp* high-risk clones, including ST11, ST147, and ST307 in human infections (34), particularly carrying the *bla_CTX-M-15_* gene (35). However, less is known about the ESBL-*Kp* STs circulating in animals and environmental sources (36). In Peru, previous genomic surveillance in the southern region revealed that all multidrug-resistant *Kp* isolates recovered from three hospitals between 2022 and 2023 were ESBL producers, predominantly belonging to the high-risk pandemic clones ST147, ST37, and ST629, and carrying *bla_CTX-M_*, *bla_SHV_*, and *bla_TEM_* alleles (37). Furthermore, the emergence of ST307 carrying the *bla_CTX-M-15_* gene was detected in a tertiary hospital (38), and ESBL-*Kp* cases were associated with neonatal sepsis (39, 40).

In 2017, we isolated the ESBL-*Kp* high-risk *Kp*clone ST307 from the feces of a pig on a farm located in coastal Peru, representing the first report of ESBL-*Kp* circulation among livestock in the country (41). Moreover, farms in this region are characterized by a high prevalence of fecal carriage of ESBL-producing *Escherichia coli* in both cows and pigs (42). However, to our knowledge, no study has simultaneously investigated the circulation of ESBL-*Kp* among humans, animals, and environmental sources in Peru, particularly in rural settings.

The aim of this study was therefore to assess the occurrence of ESBL-*Kp* across humans, domestic animals, and water samples from small-scale farms in rural Peru. We further performed whole-genome sequencing to characterize the genetic determinants of resistance and to evaluate evidence of cross-species transmission.

## 2 Methodology

### 2.1 Study regions and sampling

This study was part of a larger research project focusing on understanding the circulation of ESBL-producing Enterobacterales across the human–animal–environment interface in coastal Peru, which previously reported a high prevalence of ESBL-*E. coli* in pigs (San Bartolo district) and cows (Huaura district) on small-scale farms in Lima (42). In this study, we focused on assessing the occurrence of fecal carriage of ESBL-*Kp* in farmers and animals, as well as the ESBL-*Kp* contamination in water collected from these farms. Based on our previous studies on ESBL-Enterobacteriales [48% prevalence of ESBL-*E. coli* (43)], we assumed a prevalence of ESBL-*Kp* fecal carriage of 40%, a confidence level of 90%, and a population ranging between 100,000 and 1 million animals, resulting in a total sample size of 260 livestock animals. As we aimed to collect approximately 10 livestock samples per farm (i.e., 90% probability of detecting at least one positive animal assuming a conservative 10% prevalence), we sampled 50 farms. In those farms, we aimed to collect samples from all dogs, all humans working directly with livestock, and one water sample. Dairy farms (*n* = 25) were selected as follows: 14 farms were randomly selected from a list of 100 member farms belonging to a local dairy association (the ‘Asociación de Ganaderos de la Irrigación San Felipe’) in the Vegueta district of Huaura in Lima; one farm was selected through an ongoing collaboration with the Universidad Peruana Cayetano Heredia (UPCH) in the Lurin district; and 10 farms were randomly selected from a list provided by a veterinarian working with UPCH in the Cañete district. Pig farms (*n* = 25) were randomly selected from a list of 50 farm owners in the La Chutana district of San Bartolo in Lima. Sampling was conducted between May and July 2023. A total of 652 samples were collected, including 505 fecal samples from livestock (244 cows and 261 pigs), 58 fecal samples from dogs, 39 fecal swabs from farmers who voluntarily participated, and 50 water samples (500 mL) from livestock water sources. Farm locations and details of husbandry practices (e.g., herd size, biosecurity level, antibiotic use) are provided in Salgado-Caxito et al. (42).

### 2.2 ESBL-*Kp* isolation and antimicrobial susceptibility test

Microbiological procedures to isolate ESBL-producing Enterobacterales from fecal samples were performed as described in Salgado-Caxito et al. (42). Briefly, each fecal sample was stored in Stuart transport medium, plated on MacConkey agar (Himedia®, Maharashtra, India) supplemented with 4 mg/L of cefotaxime, and incubated at 37 °C for 24–48 h. For water samples, 500 mL were filtered through a 0.45 μm pore-size membrane filter (Millipore, USA) (44). The filters were then processed using the same procedure as that used for fecal samples. From plates showing bacterial growth, we selected all morphotypes compatible with *Klebsiella* spp., which resulted in the selection of a single colony per plate.

Bacterial species identification was performed using the VITEK®2 Advanced Expert System™ (AES), according to the manufacturer’s instructions. Phenotypic ESBL production was assessed using the combination disk test with cefotaxime (30 μg) and ceftazidime (30 μg) disks, tested alone and in combination with clavulanic acid (30/10 μg), following Clinical and Laboratory Standards Institute (CLSI) guidelines. *Klebsiella pneumoniae* subsp. pneumoniae ATCC® 700,603™ was used as a positive control for ESBL production, and *Escherichia coli* ATCC® 25,922™ (Microbiologics, St. Cloud, MN, USA) was used as the negative control.

Susceptibility to antimicrobials was also evaluated using the VITEK®2 AES. The following *β*-lactam antibiotics were included in the study: ceftazidime, cefepime, piperacillin/tazobactam, ertapenem, and meropenem. We also tested the fluoroquinolone ciprofloxacin and the aminoglycoside amikacin, two antibiotic classes widely used in swine and cattle farms in Peru and other low-income countries (30, 45, 46). Minimum inhibitory concentrations (MICs) were determined using the VITEK®2 (software version 9.02) in Natural Resistance (NATR) mode, based on Clinical and Laboratory Standards Institute (CLSI) guidelines (47).

### 2.3 Whole genome sequencing (WGS)

Isolates confirmed as ESBL-*Kp* were submitted for short-read (Illumina®) genome sequencing at MicrobesNG (Birmingham, UK). DNA extraction, library preparation, genome sequencing, read trimming, assembly, and quality control were carried out by MicrobesNG (Birmingham, UK). Briefly, 5–40 μL of bacterial culture preserved in DNA/RNA Shield (Zymo Research, USA) was mixed with an extraction buffer containing lysozyme and RNase A and incubated at 37 °C for 25 min. Then, Proteinase K and a second aliquot of RNase A were added, and the incubation was repeated at 65 °C for 5 min. A DNA library was prepared using the Nextera XT Library Preparation Kit (Illumina Inc.), and short-read sequencing was performed on the Illumina NovaSeq 6,000 (Illumina, San Diego, USA) using 2 × 250 bp paired-end reads, with a minimum coverage of 30×, following the manufacturer’s protocol. Short-reads were adapter-trimmed using Trimmomatic (version 0.30) (48). Trimmed short-reads were then *de novo* assembled using SPAdes (version 3.14.1) (49), and contigs were annotated using Prokka (version 1.11) (50). Genome quality assessment of assemblies was performed with QUAST (51) and BUSCO (version 5.3.2, Enterobacterales_odb10 database) (52). Species identification and contamination screening were performed using Kraken2 (version 2.1.1) (53). Complete information about the sequencing and assembly processes, including the MicrobesNG pipeline, procedures, and software used, is publicly available at https://microbesng.com.

*In silico* analyses of the ESBL-*Kp* genomes were conducted using the Bacterial and Viral Bioinformatics Resource Center (BV-BRC^1^) (54) and the Center for Genomic Epidemiology (CGE^2^). We used BV-BRC to obtain a comprehensive genome report, including assembly statistics (completeness and contamination), genome annotations (i.e., genes related to antimicrobial resistance (AMR), drug targets, transporters, and virulence factors), and data visualization (55). All ESBL-*Kp* genomes were considered of good quality, with 97.1–98.7% consistency and 100% completeness based on BV-BRC assembly statistics (56). Mean coverage depth was 62.8 × (range: 45.3x-127x). Genome sizes averaged 5.52 Mb (range: 5.3–5.74 Mb), with contig N50 values averaging 3.57 Mb (range: 1.61–7.66 Mb), and an average GC content of 57.1%.

The BV-BRC tool was also used to predict phenotypic antibiotic resistance based on genomic features. The CGE was used to detect plasmid replicon types [PlasmidFinder v2.0.1 (57, 58)] and to identify *Kp* sequence types (STs) [MLST v2.0.9; (56, 58–63)]. The CGE tool MobileElementFinder (MGE) v1.0.3 was used to identify whether ESBL-encoding genes were associated with plasmids or chromosomes (64). AMR genes were predicted using a 95% identity threshold and a minimum coverage of 80%, while the same identity threshold with a minimum coverage of 60% was applied for plasmid detection (65, 66). For virulence genes, predictions were performed using a 95% identity threshold, the BLOSUM80 matrix, *E*-value < 0.01, and >70% coverage (67). All databases were accessed in March 2024.

**Figure 1:**
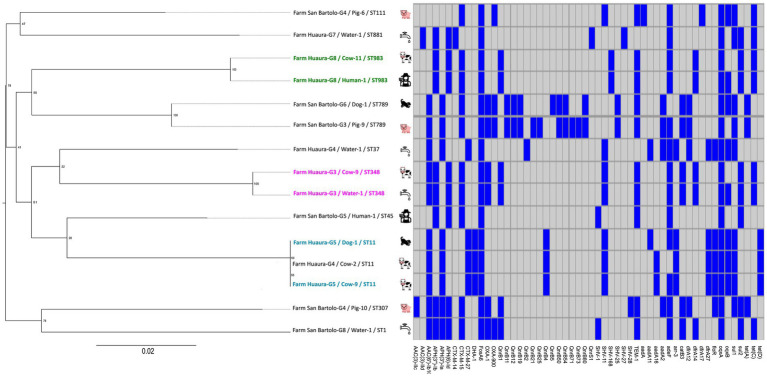
Core genome MLST (cgMLST) phylogeny and AMR gene profile of the 15 ESBL-*Klebsiella pneumoniae* isolates in rural Peru. Left: CgMLST phylogeny. Isolates sharing the same sequence type (ST) and originating from the same farm are highlighted in bold and with identical colors. The cgMLST tree was built using the PhyML+SMS tool implemented in the NGPhylogeny.fr online platform. Node labels indicate bootstrap support values, and the scale bar represents the number of substitutions per site. Figure created using FigTree v1.4.4. Right: Heatmap indicating the presence (blue) or absence (gray) of antimicrobial resistant genes (*x*-axis) for each isolate in the phylogenetic tree. Figure created using R 4.2.1.

**Figure 2:**
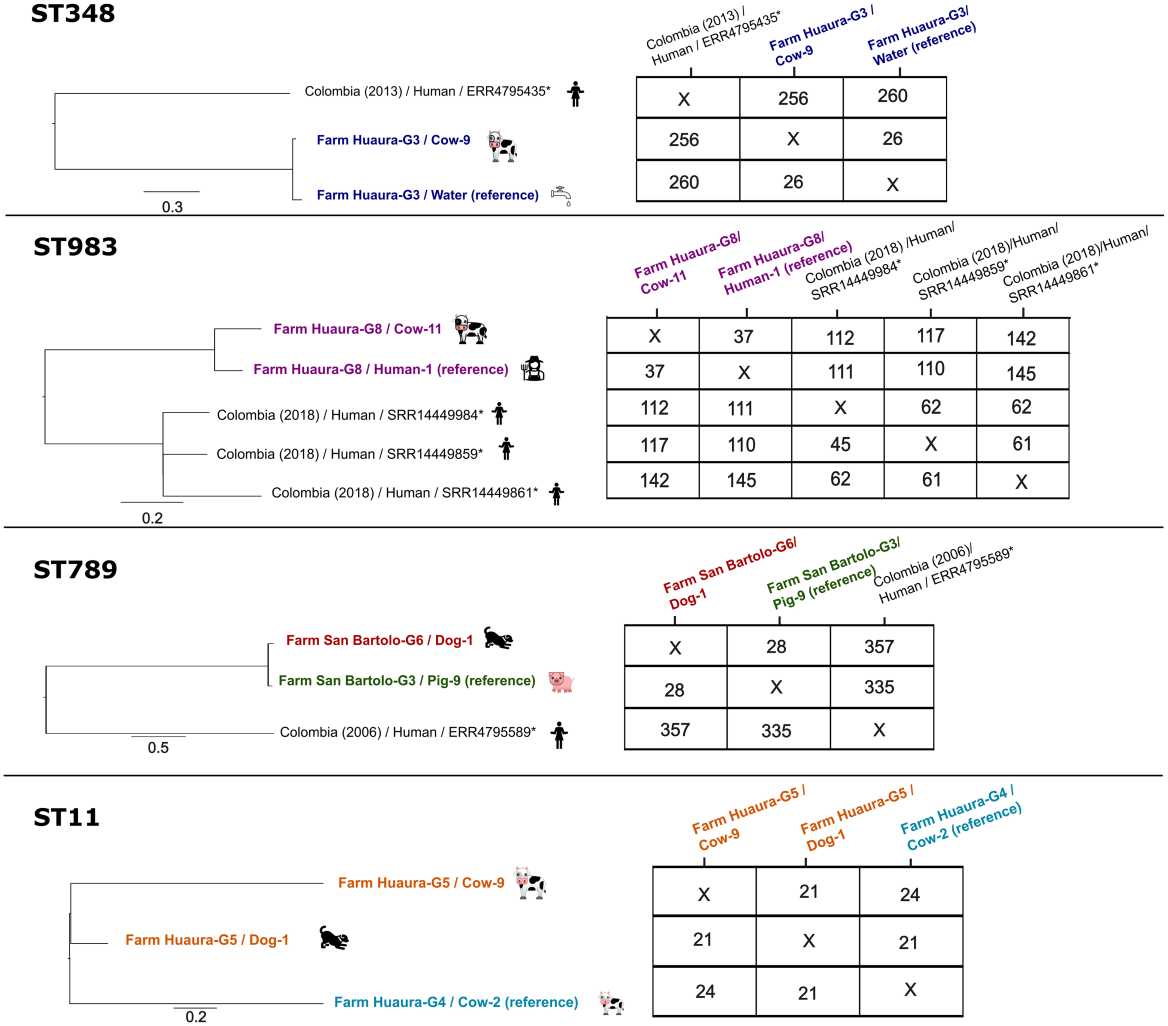
SNP-based phylogeny and pairwise SNP differences of ESBL-*Kp* isolates sharing the same ST within and across farms. Left: SNP-based phylogenetic trees were built with FastTree on the CGE platform (CSIPhylogeny) with default parameters. Colored labels indicate farm of origin of each isolate. The scale bar represents the number of substitutions per site. Right: Pairwise SNP distance matrices generated from the SNP-based phylogenetic trees shown on the left. Public Latin American genomes were included when fewer than three isolates per ST were available and were retrieved from the BIGSdb Pasteur database (https://bigsdb.pasteur.fr). Figure created using FigTree v1.4.4. and Inkscape.

To investigate the phylogenetic relationships of the ESBL-*Kp* isolates, we constructed a core genome MLST (cgMLST) phylogeny using the NGPhylogeny ‘A la carte Workflows’ platform^3^ (68). Phylogenetic trees were inferred using a maximum-likelihood approach with Smart Model Selection (PhyML + SMS), applying default parameters and 100 bootstrap replicates (Figure 1). In addition, clonal circulation was accessed by building single-nucleotide polymorphism (SNP)-based phylogenetic trees for ESBL-*Kp* isolates of the same ST using CSI Phylogeny 1.4 from the CGE (69–73). The obtained phylogenetic tree (Figure 2) was built using the altered version of FastTree with default parameters and visualized using FigTree v1.4.4. Because CSI Phylogeny requires at least three genomes to generate a SNP-based phylogeny, publicly available genomes of the same ST from South America were included when only two isolates of a given ST were identified in our dataset. These genomes were retrived from the Klebsiella Pasteur MLST database^4^. Given potential biases related to assembly differences between our sequences and publicly available assemblies, we re-assembled all genomes included in the SNP-based phylogenetic analyses on the BV-BRC platform using publicly available reads. Metadata for each genome in the phylogenetic analyses (source, year, country) are provided in Figure 2.

We also performed a SNP-based phylogenetic analysis comparing the ESBL-*Kp* ST307 isolate detected in a pig in Peru in our previous study in 2017 (41) with a newly isolated ST307 from this study. We included two other ST307 genomes, one from a human UTI in a hospital in Brazil (2016) (ERR4822787) and one from a bloodstream infection in Colombia (2014) (ERR4795646), representing the geographically closest ST307 genomes available in the database that included raw sequencing reads. In all SNP-based phylogenies, the isolate with the largest assembled genome size was used as the reference. The resulting Newick files were visualized and edited using FigTree v1.4.4^5^.

### 2.4 Ethics approval statement

This project was approved by the human and animal ethics committees (Comité Institucional de Ética en Humanos and Comité Institucional de Ética para el Uso de Animales) of the Universidad Peruana Cayetano Heredia under protocols 591–49-22 (human ethics committee) and 050–12-22 (animal ethics committee). Written informed consent was obtained from all farmers involved in this study.

## 3 Results

A total of 15 ESBL-*Kp* isolates were recovered from 652 samples screened (2.3%). ESBL-*Kp* isolates were obtained from all sampled sources, including two humans, two dogs, four cows, three pigs, three water samples from dairy farms, and one water sample from a swine farm (Table 1). Antimicrobial susceptibility tests showed that all ESBL-*Kp* isolates exhibited phenotypic resistance to ceftazidime and cefepime, and the majority were resistant to ampicillin/sulbactam (11/15) and ciprofloxacin (12/15). None of the isolates were resistant to ertapenem, meropenem, or amikacin. However, some isolates exhibited intermediate susceptibility to ampicillin/sulbactam (4/15), ertapenem (1/15), and ciprofloxacin (2/15).

**Table 1:** Source, farm of origin, and genetic profile of the 15 ESBL-*Kp* isolates from livestock farms in Peru.

Source	Farm	ST	AMR extrinsic genes	Virulence genes	Plasmid replicons
Water	San Bartolo-G8	ST1	*aac(6′)-Ib10, adeF, aph(3′)-Ia, aph(3″)-Ib, aph(6)-Id, bla_CTX-M-15_, bla_OXA-1_, bla_OXA-900_, bla_SHV-1_, bla_TEM-1_, catB3, dfrA14, fosA6, oqxA, qnrB1, sul2, tet(C)*	*entD, iroE, iutA, rfbA, rfbB, rfbD, vipB/tssC*	IncFIB(K)
Cow	Huaura-G4	ST11	*aac(6′)-Ib10, aadA16, aph(3′)-Ia, arr-3,* *bla_CTX-M-27_, bla_DHA-1_, bla_SHV-11_, dfrA27,* *floR, fosA6, oqxA, oqxB, qnrB4, sul1,* *tet(D)*	*iroE, iutA, rfbK1, vipB/tssC*	Col440I, IncFIB(K), IncFIB(pKPHS1), IncFII(K), repB(R1701)
Cow	Huaura-G5	ST11	*aac(6′)-Ib10, aadA11, adeF, aph(3′)-Ia,* *arr-3, bla_CTX-M-27_, bla_DHA-1_, bla_SHV-11_, dfrA27, floR, fosA6, oqxA, oqxB, qnrB4,* *sul1, tet(D)*	*iroE, iutA, rfbK1, vipB/tssC*	Col(pHAD28), IncFIB(K), IncFIB(pKPHS1), IncFII(K), repB(R1701)
Dog	Huaura-G5	ST11	*aac(6′)-Ib10, aadA16, adeF, aph(3′)-Ia,* *arr-3, bla_CTX-M-27_, bla_DHA-1_, bla_SHV-11_, dfrA27, floR, fosA6, oqxA, oqxB, qnrB4,* *sul1, tet(D)*	*iroE, iutA, rfbK1, vipB/tssC*	Col(pHAD28), IncFIB(K), IncFIB(pKPHS1), IncFII(K), repB(R1701)
Water	Huaura-G4	ST37	*aac(6′)-Ib10, aadA11, aadA2, adeF, aph(3′)-Ia, arr-3, bla_CTX-M-27_, bla_SHV-11_, dfrA12, dfrA27, floR, fosA6, oqxA, oqxB, qnrB2, sul1*	*entD, impA/tssA, iroE, iutA*	IncFIA(HI1), IncFIB(K)(pCAV1099-114), IncR, repB(R1701)
Human	San Bartolo-G5	ST45	*adeF, aph(3″)-Ib, aph(6)-Id, bla_CTX-M-15_, bla_SHV-1_, bla_TEM-1_, fosA6, oqxA, sul2*	*entD, fyuA, impA/tssA, iroE, irp1, irp2, iutA, rfbA, rfbB, rfbD, rfbK1, vipB/tssC, ybtA, ybtE, ybtP, ybtQ, ybtS, ybtT, ybtU, ybtX*	IncFIB(K)
Pig	San Bartolo-G4	ST111	*aadA, adeF, bla_CTX-M-15_, bla_OXA-900_, bla_SHV-11_, bla_TEM-1_, dfrA17, fosA6, oqxA, oqxB, sul1, tet(C)*	*entD, fyuA, iroE, irp1, irp2, iutA, rfbA, rfbB, rfbD, vipB/tssC, ybtA, ybtE, ybtP, ybtQ, ybtS, ybtT, ybtU, ybtX*	IncFIB(K)
Pig	San Bartolo-G4	ST307	*aac(3)-IIc, aac(6′)-Ib10, aadA2, adeF,* *aph(3′)-Ia, aph(3″)-Ib, aph(6)-Id, bla_CTX-M-15_, bla_OXA-1_, bla_SHV-28_, bla_TEM-1_, catB3, dfrA12, floR, fosA6, oqxA, qnrB1, sul1, sul2, tet(A)*	*iroE, iutA, rfbA, rfbB, rfbD, vipB/tssC*	IncFIB(K)
Cow	Huaura-G3	ST348	*aac(6′)-Ib10, adeF, aph(3″)-Ib, aph(6)-Id, bla_CTX-M-15_, bla_OXA-1_, bla_SHV-11_, bla_TEM-1_,* *catB3, dfrA14, fosA6, oqxA, oqxB, qnrB1, sul2, tet(C)*	*fyuA, impA/tssA, iroE, irp1, irp2, iutA, rfbA, rfbB, rfbD, rfbK1, vgrG/tssI, vipB/tssC, ybtA, ybtE, ybtP, ybtQ, ybtS, ybtT, ybtU, ybtX*	IncFIB(K), IncFII(K)
Water	Huaura-G3	ST348	*aac(6′)-Ib10, adeF, aph(3″)-Ib, aph(6)-Id, bla_CTX-M-15_, bla_OXA-1_, bla_SHV-11_, bla_TEM-1_, catB3, dfrA14, fosA6, oqxA, qnrB1, sul2, tet(C)*	*fyuA, impA/tssA, iroE, irp1, irp2, iutA, rfbA, rfbB, rfbD, rfbK1, vgrG/tssI, vipB/tssC, ybtA, ybtE, ybtP, ybtQ, ybtS, ybtT, ybtU, ybtX*	IncFIB(K), IncFII(K)
Pig	San Bartolo-G3	ST789	*aac(6′)-Ib10, aadA2, aph(3′)-Ia, bla_CTX-M-15_, bla_OXA-1_, bla_OXA-900_, bla_SHV-25_, bla_TEM-1_, catB3, dfrA12, fosA6, oqxA, oqxB, qnrB11, qnrB12, qnrB19, qnrB5, qnrB50, qnrB54, qnrB80, sul1, tet(A)*	*entD, iroE, irp1, rfbA, rfbB, rfbD, vipB/tssC*	Col(pHAD28), IncFIB(K), IncFII(K)
Dog	San Bartolo-G6	ST789	*aac(6′)-Ib10, aadA2, adeF, aph(3′)-Ia, bla_CTX-M-15_, bla_OXA-1_, bla_OXA-900_, bla_SHV-25_, bla_TEM-1_, catB3, dfrA12, fosA6, oqxA, qnrB11, qnrB12, qnrB19, qnrB21, qnrB25, qnrB50, qnrB54, qnrB71, qnrB73, qnrB80, sul1, tet(A)*	*entD, iroE, iutA, rfbA, rfbB, rfbD, vipB/tssC*	Col(pHAD28), IncFIB(K), IncFII(K)
Water	Huaura-G7	ST881	*aac(3)-IId, adeF, aph(3″)-Ib, aph(6)-IdV, bla_CTX-M-14_, bla_SHV-27_, fosA6, oqxA, qnrS1, sul1, sul2, tet(C)*	*iroE, iutA, rfbA, rfbB, rfbD, rfbK1, vipB/tssC*	IncFIB(K)
Cow	Huaura-G8	ST983	*adeF, aph(3″)-Ib, aph(6)-Id, bla_CTX-M-15_, bla_SHV-168_, bla_TEM-1_, dfrA14, fosA6, oqxA, qnrB1, sul2, tet(C)*	*entD, fyuA, iroE, irp1, irp2, iutA, vipB/tssC, ybtA, ybtE, ybtP, ybtQ, ybtS, ybtT, ybtU, ybtX*	IncFIB(K)
Human	Huaura-G8	ST983	*adeF, aph(3″)-Ib, aph(6)-Id, bla_CTX-M-15_, bla_SHV-168_, bla_TEM-1_, dfrA14, fosA6, oqxA, oqxB, qnrB1, sul2, tet(C)*	*entD, fyuA, iroE, irp1, irp2, iutA, vipB/tssC, ybtA, ybtE, ybtP, ybtQ, ybtS, ybtT, ybtU, ybtX*	IncFIB(K)

Only extrinsic AMR genes are listed, and virulence genes are shown only when they are absent in at least one isolate (i.e., not present in 100% of isolates).

### 3.1 Antimicrobial resistance genes

Genomic analyses identified 94 unique AMR genes. Of these, 28 (30%) were classified as intrinsic, 54 (57%) as extrinsic (acquired), and 12 (13%) as indeterminate, mostly corresponding to chromosomal mutations or resistance determinants not clearly attributable to horizontal transfer. The distribution of extrinsic AMR genes differed across sources. Dog isolates exhibited the highest number of extrinsic AMR genes per genome (16 and 25 genes), followed by pigs (12, 20, and 22 genes), cows (12 and 16 genes), and water-derived isolates (12 to 17 genes). In contrast, the two human isolates harbored only 9 and 13 genes. For the 7 antibiotics tested, the predicted resistance from the genomes using BV-BRC agreed with the phenotypic resistance obtained through the VITEK®2 AES system in 89.8% of all combinations (Supplementary Table S2).

*β*-lactam resistance genes detected included variants of *bla_CTX-M_*, *bla_SHV_*, *bla_TEM_*, *bla_OXA_*, and *bla_DHA_*, which together accounted for resistance to ceftazidime, cefepime, and ampicillin/sulbactam. ESBL-encoding genes were detected in all isolates including *bla_CTX-M-15_* in 10 isolates, *bla_CTX-M-27_* in four isolates, and *bla_CTX-M-14_* in one isolate. Narrow-spectrum β-lactamases were also common, including *bla_TEM-1_* (10 isolates), *bla_OXA-1_* (6 isolates), and *bla_OXA-900_* (4 isolates), as well as six distinct SHV alleles (*bla_SHV-11_* in seven isolates, *bla_SHV-168_* in two isolates, *bla_SHV-25_* in two isolates, *bla_SHV-1_* in two isolates, *bla_SHV-27_* in one isolate, and *bla_SHV-28_* in one isolate). The plasmid-mediated AmpC gene *bla_DHA-1_* was detected in the three ST11 isolates. Common resistance genes to other antibiotic classes included aminoglycoside-modifying enzymes such as *aadA*, *aph(3′), aph(6)-Id,* and *aac(6′)-Ib*; plasmid-mediated quinolone resistance genes (*qnrB*); sulfonamides (*sul1* or *sul2*); fosfomycin (*FosA6*); tetracycline (*tet(A)*, *tet(C)*, or *tet(D)*); trimethoprim (*dfrA12*, *dfrA14, dfrA17*, or *dfrA27*). In contrast, genes conferring resistance to phenicol (*floR* in 5 isolates) were less common. All AMR and virulence genes detected in these isolates are provided in Supplementary Table S1.

### 3.2 Virulence-associated genes

A total of 71 non-redundant virulence-associated genes were identified across the 15 ESBL-*Kp* genomes. However, none of the ESBL-*Kp* isolates carried core hypervirulence regulators such as *iucA, iroB, peg-344, rmpA*, and *rmpA2*. Virulence genes detected were associated with siderophore-mediated iron acquisition systems (26 genes, 37%, e.g., *iroE*) and adhesion factors (19 genes, 27%, e.g., *fimK* and *mrkI*), followed by capsule/LPS-related loci (7 genes, 10%) and Type VI secretion system (T6SS) components (12 genes, 17%, e.g., *tssG*, *vasE/tssK*). Water-derived isolates carried 54–70 virulence-associated genes, cow isolates 54–70, pig isolates between 56 and 68, and dog isolates 54–57. Virulence gene profiles were largely similar across sampled sources. The presence of each AMR and virulence gene was used to compare isolates belonging to the same ST (Figure 1).

### 3.3 Plasmid replicons

The PlasmidFinder tool predicted nine different plasmid replicons in the ESBL-*Kp* isolates, including Col(pHAD28), Col440I, IncFIA(HI1), IncFIB(K), IncFIB(K)(pCAV1099-114), IncFIB(p*KP*HS1), IncFII(K), IncR, and repB(R1701). The plasmid replicon IncFIB(K) was present in all genomes. IncFII(K) was also frequently identified (7/15) and occurred exclusively in co-occurrence with IncFIB(K). Smaller plasmids such as Col(pHAD28) and repB(R1701) were detected in four isolates, while IncR and Col440I were detected in only one genome each (Table 1). The MGE tool identified that only the ESBL gene *bla_CTX-M-27_* was located in the same contig as a plasmid, including three associations with plasmid replicon IncFII(K), and one association with the plasmid replicon IncFIA(Hl1), together with the *floR* gene. The genes *qnrB5* and *qnrB81* were associated with plasmid replicon Col440I in one isolate, while *bla_OXA-1_* was associated with IncFII(K) in one isolate.

### 3.4 Sequence types

The CGE MLST tool identified ten different *K. pneumoniae* sequence types (STs), including ST1, ST11, ST37, ST45, ST111, ST307, ST348, ST789, ST881, and ST983 (Table 1). Four STs (ST348 (n = 2), ST983 (n = 2), ST789 (n = 2), and ST11 (n = 3)) were found in more than one source (Table 1 and Figure 1). No STs were shared between dairy and swine farms. The cgMLST phylogeny showed that isolates with the same ST were closely related, whether they were recovered from the same farm (ST348, ST983, ST11) or from different farms (ST789, ST11) (Figure 1). However, several nodes on the cgMLST phylogenetic tree had a low bootstrap value, limiting robust inferences of relationships across different STs.

SNP analyses and the comparison of genetic profiles (including AMR genes, virulence genes, and plasmid replicons) showed a high degree of genomic similarity among isolates belonging to the same *Kp* ST (Figure 2). On farm Huaura-G8, the two ST983 isolates (one recovered from a human and one from a cow) differed by 37 SNPs and shared identical virulence gene and plasmid replicon profiles, differing only by a single AMR gene (*oqxB*). Similarly, on farm Huaura-G3, the two ST348 isolates from a cow and the water source differed by 26 SNPs and had nearly identical genetic profiles, again differing only by the *oqxB* gene. For ST789, isolates recovered from a pig (Farm San Bartolo-G3) and a dog (Farm San Bartolo-G6) differed by 28 SNPs. These isolates carried the same plasmid replicons but differed by two virulence genes (*irp1*, *iutA*) and seven AMR genes, including five *qnrB* alleles, *adeF*, and *oqxB*. All three ST11 isolates differed by 24 SNPs or less, and all harbored the AmpC *β*-lactamase gene *bla_DHA-1_*. The ST11 isolated from a dog on farm Huaura-G5 differed by 21 SNPs from the ST11 isolate recovered from a cow on the same farm, and by 21 SNPs from a ST11 isolate obtained from one cow of another farm (Huaura G4). The two ST11 isolates from cows of different farms differed by 24 SNPs. Across the ST11 isolates, gene content variation was limited, with differences of no more than two AMR genes and two virulence genes. The two ST11 isolates from the same farm (Huaura G5) carried the same five plasmid replicons (Col(pHAD28), IncFIB(K), IncFIB(p*KP*HS1), IncFII(K), repB(R1701)). In contrast, the remaining ST11 isolate from the other farm carried Col440I instead of Col(pHAD28) (Table 1).

The SNP phylogeny of the four ESBL-*Kp* ST307 isolates revealed pairwise SNP differences ranging from 50 to 300. In particular, the ST307 isolate identified in this study differed by 251 SNPs from the ST307 isolate previously recovered in 2017 from a pig in the same region.

## 4 Discussion

The dynamics of ESBL-*Klebsiella pneumoniae* (ESBL-*Kp*) in the community remain poorly understood, despite increasing evidence of community-acquired infections caused by these bacteria. In particular, the extent of ESBL-*Kp* circulation across the human–animal–environment interface in LMICs, including those in Latin America, remains unknown. In this study, we detected ESBL-*Kp* across all sampled source types in a rural setting in coastal Peru, including humans, pigs, cows, dogs, and water. This finding suggests that ESBL-*Kp* circulates broadly across sources, despite a low overall isolation frequency (2.3%). Genomic analyses revealed a diverse population structure, comprising 10 different ESBL-*Kp* sequence types, with the majority of isolates (10/15) carrying the ESBL gene *bla_CTX-M-15_*. The highly genomic similarity and closely related genetic profiles observed among the four STs detected across different host species and environmental sources suggest clonal exchanges of ESBL-*Kp* at this farmer–animal–water interface.

ESBL-*Kp* is of critical importance to public health because of the difficulties in treating infections caused by this bacterium (74). The proportion of hospital- and community-acquired infections caused by ESBL-*Kp* is relatively low (<5%) among *Kp* infections (75, 76). Therefore, a low fecal carriage rate of ESBL-*Kp* in human and animal populations is also expected (19, 26). Our findings support this expectation: only a small proportion of human and animal fecal samples (<3%) carried ESBL-*Kp*. Despite this low detection rate, ESBL-*Kp* was found across all sampled source types (humans, livestock, dogs, and water) and across multiple farms and localities. This widespread distribution highlights the need for a better understanding of the selective pressures and circulation pathways that maintain these bacteria in this rural environment. The absence of apparent clinical signs in the sampled animals and humans, together with the absence of the five genes (*iucA, iroB, peg-344, rmpA*, and *rmpA2*) associated with hypervirulence in the detected bacteria (77), suggests that these strains are classical, likely commensal *K. pneumoniae*.

The molecular mechanisms driving the spread of ESBL-*Kp* were first attributed to SHV- and TEM-type β-lactamases genes in the Americas, followed by the expansion of CTX-M-encoding genes, particularly *bla_CTX-M-15_* in North America (28, 78). Our genomic analyses revealed a high diversity of ESBL-*Kp* STs including high-risk global clones ST307 and ST11 (32, 33), and three CTX-M-encoding alleles, confirming the predominant circulation of *bla_CTX-M-15_*. A high clonal diversity of ESBL-*Kp* has also been described in hospital settings (79) and in community-acquired infections (31, 80). In our study, only the gene *bla_CTX-M-27_* was detected on the same contig as a plasmid, suggesting that *bla_CTX-M-15_* and *bla_CTX-M-14_* may be chromosomally located. However, long-read sequencing would be needed to establish the exact location of these genes and to confirm this hypothesis, particularly since *bla_CTX-M-15_* is often carried in plasmids (81, 82). The ESBL-encoding genes *bla_CTX-M-15_* and *bla*_CTX-M-14_, and the narrow-spectrum *β*-lactamases *bla_SHV-1_*, *bla_SHV-11_*, and *bla_SHV-27_*, have been previously reported in *Kp* from hospitals in Peru (37, 39, 40, 83). This indicates that multiple molecular mechanisms responsible for ESBL-*Kp* are selected in both hospital and community settings, including rural areas. We also found the high-risk clone ESBL-*Kp* ST307 in a pig carrying the same ESBL-encoding genes as an ST307 previously isolated from a pig in the same area in 2017 (41). Although the genomic differences between these isolates (i.e., 251 SNPs) do not support them as being the same clone, these findings suggest that ESBL-*Kp* has been circulating within this animal population for several years rather than representing a recent introduction.

Circulation of ESBL-Enterobacterales across the components of the human–animal–environment interface can have important consequences for both human and animal health. However, such transmission is often difficult to detect, since most studies do not sample multiple sources at the same time and location (84). In this study, we identified four ESBL-*Kp* STs present in more than one source. Phylogenomic analyses of these STs were consistent with potential cross-species clonal circulation events. In particular, we detected a farmer and a cow from the same farm harboring almost identical ESBL-*Kp* ST983 genomes and genetic profiles *Kp*, suggesting either cross-species transmission or contamination from a common source. This result is in line with previous evidence of human–dog transmission in household settings in high-income countries (85), but contrasts with findings from Guadeloupe island, where limited cross-species transmission was observed (86). We also detected very similar ESBL-*Kp* ST11 isolates collected from a dog and a cow from the same farm, as well as from a cow on a different farm. This suggests potential circulation of the same ESBL-*Kp* clone between farms, possibly mediated by the movement of animals, people, or contaminated materials. Together, these findings highlight the need to identify practices and sanitary conditions that facilitate bacterial transmission between humans and their livestock in rural settings.

The environment of humans and animals, including water sources, can also participate in the circulation of ESBL-*Kp* (87, 88). However, the role of water in the spread of ESBL-*Kp* in rural environments in Latin America remains poorly studied, particularly in Peru (87). We detected ESBL-*Kp* in the water sources of four farms (one swine farm and three dairy farms). These isolates belonged to four different STs (ST1, ST348, ST881, and ST37). ESBL-*Kp* ST348 was nearly identical to an isolate from a cow collected on the same farm. The results of this study support the need to design One Health-oriented interventions to interrupt human–animal–environment circulation, including measures to reduce water contamination by ESBL-*Kp*.

Our study represents a first step in understanding ESBL-*Kp* circulation in Peruvian communities. However, several limitations should be addressed by future research. First, the overall ESBL-*Kp* genetic diversity circulating at this interface is likely underestimated, particularly since we relied on a single-colony selection per host. Although a low within-host diversity of ESBL-*Kp* has been estimated in humans (89), studying within-host diversity could allow a better understanding of the full extend of ESBL-*Kp* diversity in this interface, particularly for animal or water samples. Second, although this study included a large number of sample, its cross-sectional design does not allow the evaluation of temporal trends, which could contribute to inferring directionality of bacterial circulation across hosts and environments. Longitudinal studies however, are difficult to conduct and require sustained funding and long-term farmer engagement. Finally, future studies could further characterize the molecular mechanisms involved in the spread of the detected ESBL-encoding genes. For example, the use of long-read whole-genome sequencing would allow determining whether some of the detected genes (e.g., *bla_CTX-M-15_*) are located on plasmids.

## Data Availability

Sequences are available at the European Bioinformatics Institute (EMBL-EBI) using the project accession number PRJEB107091.

## Footnotes

1
https://www.bv-brc.org

2
http://www.genomicepidemiology.org/

3
http://NGPhylogeny.fr

4 https://bigsdb.pasteur.fr/, data submitted up to 2024-12-31

5
https://tree.bio.ed.ac.uk/software/figtree/
